# Navigating the Nexus: A Rare Case of Choledochal Cyst With Concomitant Pancreas Divisum

**DOI:** 10.7759/cureus.63964

**Published:** 2024-07-06

**Authors:** Aldrin L Myrthong, Abhinav C G, Rihan Rashid, Vinayak Venu, Vismaya Kb

**Affiliations:** 1 Department of General Surgery, Grant Government Medical College and Sir JJ Group of Hospitals, Mumbai, IND

**Keywords:** pancreas divisum, coexistence of choledochal cyst and pancreas divisum, management of coexistent choledochal cyst and pancreas divisum, rare biliary tree anomalies, biliary tree anomalies, excision of choledochal cyst, choledochal cyst

## Abstract

Choledochal cysts (CCs) are quite rare and are characterized by anomalous dilations of the biliary tree, mostly due to anomalous pancreaticobiliary junction (APBJ). A less frequent congenital anomaly due to incomplete fusion of pancreatic ducts, pancreas divisum (PD) can complicate the clinical course of CC. Although rare, the coexistence of CC and PD presents significant clinical challenges. With very few documented cases globally, our experience with this case adds to our understanding of this unique condition. This report aims to highlight the complex relationship between these anomalies and underscores the need for heightened clinical awareness and comprehensive management strategies to improve patient outcomes.

We present the case of a 27-year-old female patient who was diagnosed with type 1 CC with concomitant PD after recurrent pancreatitis and multiple biliary interventions. Her choledochal cyst was excised with Roux-en-Y hepaticojejunostomy (RYHJ). Histopathological examination confirmed CC with evidence of chronic inflammatory changes but no malignancy. The preoperative hospital stay was three days with an operative duration of 150 minutes and intraoperative blood loss of 210 mL. Postoperatively, the patient was discharged on day 5. The pain score as per the Visual Analog Scale (VAS) was 2 on the day of discharge. The patient was started on diet on postoperative day (POD) 3. The abdominal drains were removed on POD 4 (subhepatic) and POD 5 (pelvic). Sutures were removed on POD 10, with follow-up for two years with no recurrence of similar complaints.

This case illustrates the diagnostic challenge of synchronous CC and PD and elaborates on the role of extensive imaging modalities in guiding management decisions. The surgical approach remains the foremost for CC; preventing complications in the form of cholangitis and malignancy is the mainstay of treatment. The present report is an addition to the existing literature on the management of complex biliary anomalies and places special emphasis on the need for a multidisciplinary approach with individualized treatment strategies in such rare clinical scenarios.

Further studies are required to clarify pathophysiological mechanisms linking CC and PD, with the need for better therapeutic strategies toward the optimization of patient outcomes. More studies with robust data are necessary to draw better conclusions.

## Introduction

Choledochal cysts (CCs) are abnormal cystic dilations within the biliary tree, primarily affecting children under 10 [1]. The exact cause is unknown but often associated with anomalous pancreaticobiliary duct junction (APBDJ) and an elongated common channel. This theory suggests reflux of pancreatic enzymes into the bile duct, triggering inflammation and ductal dilation. The reflux may extend into the pancreatic duct, leading to pancreatitis [1].

Pancreas divisum (PD) is the most common congenital pancreatic anomaly, resulting from incomplete fusion of pancreatic buds. Although rare, PD can cause elevated ductal pressures and recurrent pancreatitis [2].

Although rare, the coexistence of CC and PD presents significant clinical challenges [2]. With fewer than 10 documented cases globally, our experience with this case adds to our understanding of this unique condition. This report highlights the complex relationship between these anomalies and underscores the need for heightened clinical awareness and comprehensive management strategies to improve patient outcomes.

## Case presentation

Clinical presentation

A 27-year-old female presented with a six-month history of gradual onset, intermittent, severe pain in the right upper abdomen, relieved with medication. She also reported occasional non-bilious, non-projectile vomiting, without fever or jaundice. The patient has a medical history of diabetes mellitus managed with oral hypoglycemic agents and a history of multiple procedures including endoscopic retrograde cholangiopancreatography (ERCP) with sphincterotomy and stenting of the common bile duct (CBD) six months ago, laparoscopic cholecystectomy three months ago, and stent removal after four weeks. Multiple episodes of acute pancreatitis occurred within the past year.

Examination and investigations

Upon examination, no icterus was noted, and the abdomen was soft and non-tender, with no palpable masses. Blood investigations are summarized in Table 1.

**Table 1:** Summary of blood investigations WBC: white blood cell count, SGOT: serum glutamate oxaloacetate transaminase, AST: aspartate transaminase, SGPT: serum glutamate pyruvate transaminase, ALT: alanine aminotransferase Reference ranges [3]

Investigation	Result	Reference range [3]	Full form of unit
Hemoglobin	11.7 g/dL	12-16 g/dL (male), 14-17 g/dL (female)	Grams per deciliter (g/dL)
WBC	9,000 cells/mm³	4,500-11,000 cells/mm³	Cells per cubic millimeter (cells/mm³)
Platelet count	315,000/mm³	150,000-450,000/mm³	Per cubic millimeter (mm³)
SGOT/AST	45 U/L	10-40 U/L	Units per liter (U/L)
SGPT/ALT	39 U/L	7-56 U/L	Units per liter (U/L)
Total bilirubin	1.7 mg/dL	0.3-1 mg/dL	Milligrams per deciliter (mg/dL)
Direct bilirubin	1 mg/dL	0.1-0.3 mg/dL	Milligrams per deciliter (mg/dL)
Indirect bilirubin	0.7 mg/dL	0.2-0.7 mg/dL	Milligrams per deciliter (mg/dL)
Alkaline phosphatase	316 U/L	30-120 U/L	Units per liter (U/L)
Amylase	82.6 U/L	25-125 U/L	Units per liter (U/L)
Lipase	171.3 U/L	13-95 U/L	Units per liter (U/L)
Sodium	136.8 mmol/L	136-145 mmol/L	Millimoles per liter (mmol/L)
Potassium	4.1 mmol/L	3.5-5.0 mmol/L	Millimoles per liter (mmol/L)
Creatinine	0.9 mg/dL	0.7-1.3 mg/dL (male), 0.5-1.1 mg/dL (female)	Milligrams per deciliter (mg/dL)
Urea	21 mg/dL	8-20 mg/dL	Milligrams per deciliter (mg/dL)

Imaging findings

**Figure 1:**
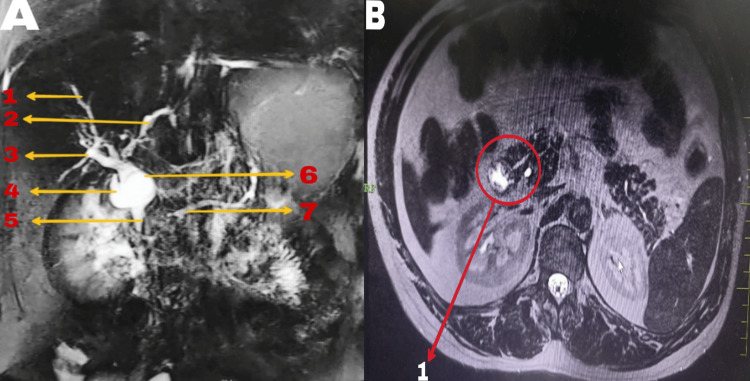
MRCP image showing biliary anatomy (A) and MRCP coronal section showing a separate opening of the pancreatic duct into the duodenum suggestive of drainage into the minor duodenal papilla (B) MRCP: magnetic resonance cholangiopancreatography, CBD: common bile duct, MPD: main pancreatic duct A1: right hepatic duct, A2: left hepatic duct, A3: cystic duct stump, A4: type 1 choledochal cyst, A5: tapered distal CBD, A6: proximal CBD, A7: mildly dilated MPD, B1: pancreas divisum

Ultrasonography revealed a dilated common bile duct (CBD) at the porta hepatis measuring 1.4 cm, with the gallbladder not visualized. Mild hepatomegaly and mild central intrahepatic biliary radicle dilatation were observed. Magnetic resonance cholangiopancreatography (MRCP) showed a fusiform dilatation of the proximal CBD with smooth tapering of the distal CBD, mild central and peripheral intrahepatic biliary radicle dilatation, and a 4.8 mm stump of the cystic duct. Borderline dilated main pancreatic duct (MPD) with pancreas divisum was noted. The CBD measured 16 mm at the porta and 4.3 mm at the distal end, suggesting features consistent with type 1 choledochal cyst and distal CBD stricture with pancreas divisum (Figure 1).

Surgical procedure

**Figure 2:**
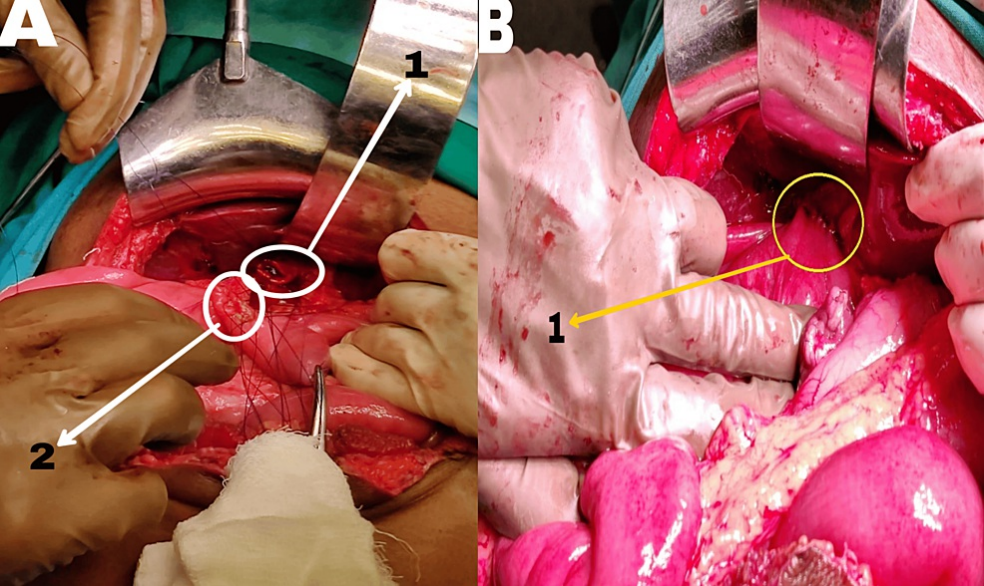
Before Roux-en-Y hepaticojejunostomy (A) and after Roux-en-Y hepaticojejunostomy (B) A1: cut end of the hepatic duct, A2: jejunal site for anastomosis, B1: hepaticojejunostomy site

Considering the fact that the patient had already undergone ERCP with sphincterotomy of the minor papilla, the current symptomatology of the patient was attributed more to a choledochal cyst than to pancreas divisum. Thus, a surgical procedure was planned for the patient based on magnetic resonance cholangiopancreatography (MRCP) findings. Choledochal cyst excision with Roux-en-Y hepaticojejunostomy (RYHJ) was undertaken. A 15-18 cm midline incision was made, revealing dense omental adhesions in the gallbladder fossa. Careful exploration identified a type 1 choledochal cyst of the common bile duct (CBD), approximately 2 cm in size. Meticulous dissection was performed to separate the choledochal cyst from the portal vein and inferior vena cava. The upper and lower limits of the cyst were identified. All major vessels, including the gastroduodenal artery, right hepatic artery, and common hepatic artery, were identified and carefully protected throughout the procedure. The choledochal cyst was excised. A loop of jejunum was transected approximately 30-40 cm away from the duodenojejunal flexure, and the loop was brought up for anastomosis with the remaining common hepatic duct. Finally, a jejuno-jejunal anastomosis was performed to maintain the continuity of the bowel (Figure 2). Then, 32 French (Fr) abdominal drains were placed in subhepatic and pelvic regions. Some of the intraoperative and postoperative parameters are summarized in Table 2 and Table 3, respectively.

**Table 2:** Intraoperative parameters

Intraoperative parameter	
Duration	150 minutes
Blood loss	210 mL
Complications	None
Preoperative hospital stay	3 days

**Table 3:** Postoperative parameters The patient was discharged on POD 5. Thus, the duration of the hospital stay was five days. All sutures were removed on POD 10. The patient was followed up weekly for one month, monthly for three months, and three monthly for one year. There were no recurrences of similar complaints in the follow-up period. POD: postoperative day, VAS: Visual Analogue Scale

POD	Pain as per VAS	Pelvic drain output over 24 hours	Subhepatic drain output over 24 hours	Diet
POD 1	4	100 cc serosanguinous	50 cc serosanguinous	Ryle's tube removed
POD 2	3	50 cc serous	30 cc serous	Liquid diet - passing flatus
POD 3	3	30 cc serous	20 cc serous	Soft diet - passing flatus
POD 4	3	20 cc serous	10 cc serous - drain removed	Soft diet - passed stools
POD 5	2	10 cc serous - drain removed	-	Full diet - passing stools

Histopathological examination

**Figure 3:**
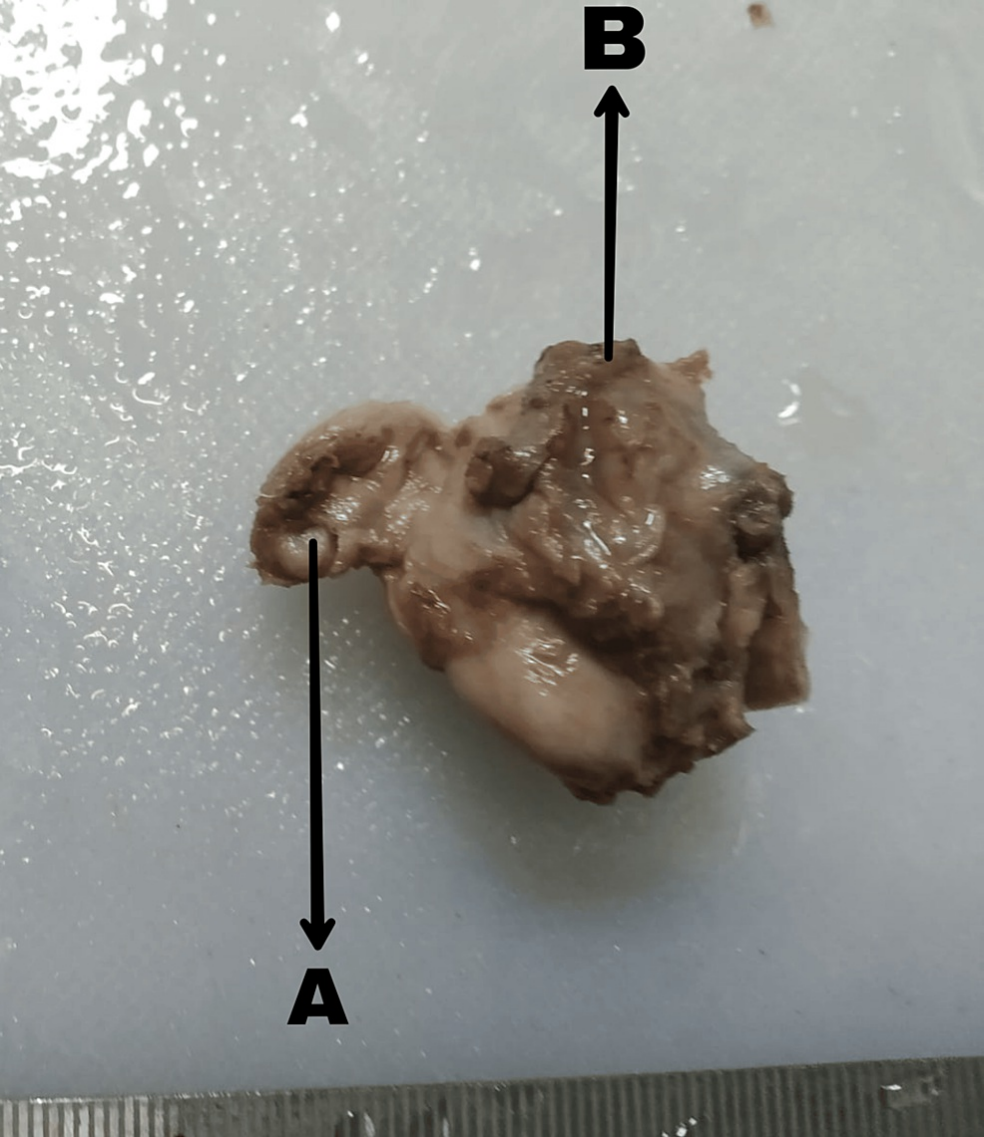
Excised biliary cyst A: cystic duct stump, B: biliary cyst

The specimen (Figure 3) along with the tapered portion of distal CBD underwent histopathological examination, revealing focal lining by columnar epithelium. A dense chronic inflammatory infiltrate, primarily comprising lymphocytes and plasma cells, formed lymphoid aggregates with prominent germinal centers. The wall structure consisted of bundles of smooth muscles along with fibrous tissue. No evidence of dysplasia, atypia, or malignancy was identified. These histopathological findings are consistent with a diagnosis of a choledochal cyst.

## Discussion

History

Biliary cysts are characterized by cystic enlargements affecting different segments of both the bile ducts outside the liver (extrahepatic) and within the liver (intrahepatic). In 1959, Alonso-Lej et al. [4] initially classified CC into three types based on the location of the bile duct dilation. It was not until 1977 that Todani et al. [5] expanded this classification by introducing two additional types of CCs. This classification, comprising five categories, is widely used in clinical practice today. However, some experts argue that each type of CC follows its distinct natural course, presents specific complications, and requires tailored management approaches.

Etiology

The precise cause of a choledochal cyst remains elusive, leading to various theories attempting to explain its origin. Among these, Babbitt's theory is the most widely accepted, proposing that the cyst stems from an abnormal junction between the pancreatic and biliary ducts, known as an anomalous pancreaticobiliary junction (APBJ). In APBJ, the connection between these ducts occurs abnormally close to the sphincter of Oddi, forming an unprotected channel where pancreatic and biliary secretions mix, triggering pancreatic enzyme activation. This process elevates pressure within the ducts, eventually resulting in dilation, inflammation, epithelial damage, dysplasia, and potentially malignancy within the biliary tree [6]. A deficiency of ganglionic cells in the distal common bile duct of affected individuals, leading to dilation of the proximal CBD similar to the pathogenesis observed in conditions such as achalasia and Hirschsprung's disease, was suggested by Kusunoki et al. [1,7,8].

For specific types of choledochal cysts, such as type 2 (true CBD diverticulum) and type 3 (choledochocele), the cause is attributed to duplications of the biliary system or adjacent structures, potentially obstructing the ampulla of Vater and leading to localized distal intramural bile duct dilation. Caroli disease, classified as a type 5 choledochal cyst, is presumed to result from a halt in the normal remodeling of ductal plates. This condition shares similarities with biliary atresia in terms of ductal plate malformation and is associated with autosomal recessive polycystic kidney disease (PKD) and, less commonly, autosomal dominant PKD [7,8].

Todani Classification

Type 1 includes choledochal cysts in a narrow sense (subtype a), segmental choledochal dilatation (subtype b), and diffuse or cylindrical dilatation (subtype c). Type 2 refers to a supraduodenal diverticulum. Type 3 encompasses choledochocele (subtype a) and diverticular choledochocele (subtype b). Type 4 comprises multiple dilatations, with subtype a involving the intrahepatic biliary tract and subtype b sparing the intrahepatic biliary tract. Type 5, also known as Caroli disease, entails multiple intrahepatic dilatations. A rare condition where only the cystic duct is dilated has been described. Some experts classify this as type 6 subtype a, while type 6 subtype b additionally involves dilatation of the common bile duct [9].

In summary, while multiple theories attempt to explain the etiology of choledochal cysts, a comprehensive understanding remains elusive, underscoring the complexity of this condition.

Epidemiology

There is a noticeable regional discrepancy in the prevalence of choledochal cysts, with a significant concentration of reported cases in Japan, where approximately two-thirds of cases in Asia are documented. This condition predominantly affects individuals of Asian descent, with an incidence of approximately one in 1,000 live births, contrasting sharply with the incidence of one in 100,000-150,000 live births observed in Western populations. Choledochal cysts of types 2 and 4 are more frequently observed in females, with a female-to-male ratio typically ranging from 3:1 to 4:1 [7,10]. Despite these observed patterns, the underlying reasons for the higher prevalence in Asian populations and the female predominance remain unclear.

Presentation

The traditional combination of jaundice, a mass in the right upper quadrant, and abdominal pain is observed in a minority of choledochal cyst patients. This symptom triad is more commonly observed in children than in adults. Additional common symptoms of choledochal cysts include cholangitis, pancreatitis, and biliary peritonitis resulting from cyst rupture.

Investigations

Ultrasound (US) serves as the primary method for initially assessing both the intrahepatic and extrahepatic biliary systems along with the gallbladder. Furthermore, US can reveal associated complications such as cystolithiasis, cholangitis, and potential malignancies. However, in adults, US may not always accurately pinpoint bile duct cysts due to the presence of various secondary malignant and benign causes for bile duct dilation [11].

Computed tomography (CT) is rarely necessary, mainly when bowel gas obscures the view of the distal common bile duct. Both US and CT excel in identifying cystic lesions in the upper right abdomen and evaluating their size and extent, although determining the biliary origin of the cyst may not always be straightforward. The presence of intrahepatic ductal dilation can provide a crucial diagnostic clue. Differential diagnoses for large cystic lesions in the porta hepatis region include gastrointestinal duplication cysts, omental cysts, mesenteric cysts, hepatic cysts, and pancreatic pseudocysts [11].

Magnetic resonance cholangiopancreatography (MRCP) and endoscopic retrograde cholangiopancreatography (ERCP) are preferred diagnostic methods for assessing biliary ductal pathology. MRCP is increasingly recognized for its high sensitivity, safety, and noninvasiveness in the preoperative detection of choledochal cysts, often supplanting the need for diagnostic ERCP in various pancreaticobiliary disorders. However, MRCP may have limitations in detecting associated ductal anomalies or small choledochocele. Additionally, it may not be suitable for pediatric patients who cannot hold their breath for the duration of the imaging sequence. In clinical practice, MRCP is typically performed before ERCP in patients suspected of having choledochal cysts based on US findings.

Management

In medical treatment, the main emphasis is on administering antimicrobial therapy for cholangitis or offering supportive care for pancreatitis, to stabilize the patient before contemplating surgical intervention. If cyst rupture transpires, drainage placement becomes essential, underscoring the atypical nature of these scenarios.

Management of type 2 and 4 CC involves complete excision of the extrahepatic bile duct cyst down to its communication with the pancreatic duct, along with cholecystectomy and restoration of bilioenteric continuity [12]. Hepatectomy is necessary for type 4A cysts with significant intrahepatic involvement likely to cause postoperative complications if left untreated. In select cases, excision of the extrahepatic duct alone may suffice, as intrahepatic duct dilation typically resolves within 3-6 months [13,14]. Both hepaticoduodenostomy and Roux-en-Y hepaticojejunostomy (RYHJ) have been described for bilioenteric reconstruction after type 1 and 4 CC resection, with RYHJ being preferred due to lower risks of gastric and biliary cancer associated with hepaticoduodenostomy [15].

Types 2 and 3 CC carry an exceedingly low risk of malignant transformation, making diverticulectomy followed by primary closure of the common bile duct (CBD) at the diverticulum neck sufficient [16]. Endoscopic sphincterotomy is appropriate for managing small choledochoceles. Transduodenal excision may be considered for large choledochoceles with complications such as gastric outlet obstruction or pancreatitis. Management of type 5 CC (Caroli disease) involves liver resection or orthotopic liver transplant (OLT) [17].

Pancreas divisum (PD) arises due to the incomplete fusion of the ductal systems of the dorsal and ventral buds during embryonic pancreatic development, typically occurring around the seventh week of pregnancy [18]. Pancreas divisum (PD) has been suggested as a potential cause of idiopathic recurrent acute pancreatitis (I-RAP) and chronic pancreatitis (CP). However, its precise clinical importance has not been definitively measurable up to this point [19].

Patients with PD experience symptoms similar to those with normal pancreatic ductal anatomy, spanning from recurring abdominal pain to acute pancreatitis and chronic pancreatitis, along with their respective complications. Asymptomatic individuals incidentally diagnosed with PD on imaging typically do not require further diagnostic or therapeutic intervention. For symptomatic patients, treatment decisions hinge on the frequency, duration, and intensity of symptoms, as well as the presence of complications [18,19]. While managing pancreatic disease, similar principles apply regardless of variations in pancreatic ductal anatomy, with a few adjustments for PD. In cases of mild or infrequent pancreatic-type abdominal pain without evidence of chronic pancreatitis on imaging, conservative approaches for symptom control are preferred, including a low-fat diet, non-narcotic pain relievers, antispasmodic medications, and pancreatic enzyme supplementation [19].

For acute pancreatitis, chronic pancreatitis, or associated complications leading to significant functional impairment, a thorough evaluation of the underlying cause is essential, including genetic testing, medication history, and metabolic assessments, to direct appropriate therapy aimed at mitigating the underlying etiology. Therapeutic intervention is typically reserved for patients with recurrent or severe acute pancreatitis or in cases of chronic pancreatitis where a modifiable target such as a stone or stricture is identified [18,19]. However, when PD coexists with chronic pancreatitis, treatment strategies align with those for patients with normal pancreatic duct anatomy. In cases where the dorsal duct is normal or mildly dilated, management primarily focuses on relieving obstruction at the minor papilla through endoscopy (e.g., ERCP) or surgery, with the choice depending on patient preferences and institutional expertise [19].

PD is often discovered incidentally during abdominal imaging for unrelated reasons, typically not warranting further evaluation or pursuit of symptoms. Contrast-enhanced magnetic resonance cholangiopancreatography (MRCP) typically has a sensitivity of 50%-70% [18,19]. However, with secretin-enhanced MRCP (S-MRCP), sensitivity can increase to 83%-86%, with a specificity of 97%-99% [19]. Endoscopic ultrasound (EUS) demonstrates high diagnostic accuracy for PD, with a sensitivity ranging from 87% to 95%. Secretin enhancement (S-EUS) provides minimal additional benefit [19,20].

Limitation

Coexistence of choledochal cyst with pancreas divisum is a very rare occurrence. Although this single-center case report adds great value to the very limited medical literature available at this point, it is necessary to carry out a well-structured multicentric research with a much higher sample size to draw better conclusions.

## Conclusions

Choledochal cysts are very rare congenital anomalies characterized by abnormal dilatation of the biliary tree, often associated with an anomalous pancreaticobiliary duct junction. The coexistence of pancreas divisum, a congenital anomaly consisting of incomplete fusion of pancreatic ducts during embryonic development with an increased risk of recurrent pancreatitis, adds to the diagnostic challenges. Therefore, such an occurrence of complications makes this case special: CC combined with PD. Surgery for excision of the cyst with restoration of normal biliary anatomy is usually required by management and is appropriately tailored to accommodate associated anatomical anomalies such as PD. Histopathological examination of the excised cyst confirmed CC and reiterated that timely surgical intervention played a critical role in mitigating its long-term complications.

Further studies are of the essence in clarifying the exact pathophysiological mechanisms underlying the coexistence of CC and PD, with the former being considered infrequent and their coexistence exerting a synergistic effect on clinical outcomes. A heightened clinical suspicion aided by previous advanced imaging techniques such as MRCP is the basis for establishing an accurate diagnosis and formulating strategies in management tailored for the patient. In other words, this case report has added further insight into the complex interplay of CC and PD management, pointing out the need for multidisciplinary approaches with special regard to tailored treatment plans for the optimization of patient outcomes. Further research is needed to refine diagnostic protocols, advance surgical techniques, and define long-term prognosis improvement for patients afflicted by these very intriguing congenital abnormalities.
